# In Situ X-ray Scattering Reveals Coarsening Rates of Superlattices Self-Assembled from Electrostatically Stabilized Metal Nanocrystals Depend Nonmonotonically on Driving Force

**DOI:** 10.1021/acsnano.3c12186

**Published:** 2024-02-06

**Authors:** Christian P. N. Tanner, James K. Utterback, Joshua Portner, Igor Coropceanu, Avishek Das, Christopher J. Tassone, Samuel W. Teitelbaum, David T. Limmer, Dmitri V. Talapin, Naomi S. Ginsberg

**Affiliations:** † Department of Chemistry, University of California, Berkeley, California 94720, United States; ‡ Department of Chemistry, James Franck Institute, and Pritzker School of Molecular Engineering, University of Chicago, Chicago, Illinois 60637, United States; § Stanford Synchrotron Radiation Lightsource, SLAC National Accelerator Laboratory, Menlo Park, California 94025, United States; ∥ Department of Physics, Arizona State University, Tempe, Arizona 85287, United States; ⊥ Chemical Sciences Division, Lawrence Berkeley National Laboratory, Berkeley, California 94720, United States; # Materials Sciences Division, Lawrence Berkeley National Laboratory, Berkeley, California 94720, United States; ∇ Kavli Energy NanoSciences Institute, University of California, Berkeley, California 94720, United States; ○ Center for Nanoscale Materials, Argonne National Laboratory, Argonne, Illinois 60517, United States; ◆ Department of Physics, University of California, Berkeley, California 94720, United States; ◇ Molecular Biophysics and Integrated Bioimaging Division, Lawrence Berkeley National Laboratory, Berkeley, California 94720, United States; ◨ Materials Sciences and Chemical Sciences Divisions, Lawrence Berkeley National Laboratory, Berkeley, California 94720, United States; ◧ STROBE, NSF Science & Technology Center, Berkeley, California 94720, United States

**Keywords:** soft condensed matter, nanocrystals, self-assembly, coarsening, X-ray scattering, *in situ* measurement

## Abstract

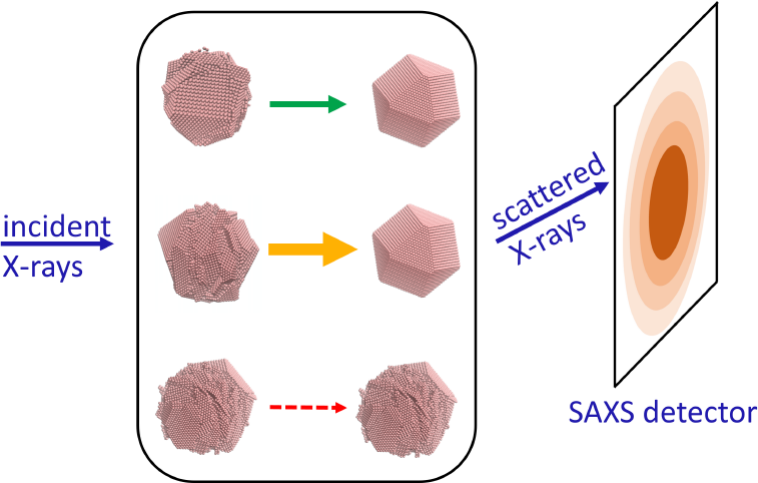

Self-assembly of colloidal nanocrystals (NCs) into superlattices (SLs) is an appealing strategy to design hierarchically organized materials with promising functionalities. Mechanistic studies are still needed to uncover the design principles for SL self-assembly, but such studies have been difficult to perform due to the fast time and short length scales of NC systems. To address this challenge, we developed an apparatus to directly measure the evolving phases *in situ* and in real time of an electrostatically stabilized Au NC solution before, during, and after it is quenched to form SLs using small-angle X-ray scattering. By developing a quantitative model, we fit the time-dependent scattering patterns to obtain the phase diagram of the system and the kinetics of the colloidal and SL phases as a function of varying quench conditions. The extracted phase diagram is consistent with particles whose interactions are short in range relative to their diameter. We find the degree of SL order is primarily determined by fast (subsecond) initial nucleation and growth kinetics, while coarsening at later times depends nonmonotonically on the driving force for self-assembly. We validate these results by direct comparison with simulations and use them to suggest dynamic design principles to optimize the crystallinity within a finite time window. The combination of this measurement methodology, quantitative analysis, and simulation should be generalizable to elucidate and better control the microscopic self-assembly pathways of a wide range of bottom-up assembled systems and architectures.

## Introduction

Colloidal nanocrystal (NC) building blocks can be used to self-assemble a variety of functional, ordered structures or superlattices (SLs)^1−11^ with potential energy and optoelectronic applications, such as solar cells,^12^ sensors,^13^ catalysts,^14^ and displays.^15,16^ In order to reliably control and direct the self-assembly of SLs, a detailed understanding of the microscopic interactions between NCs and of the thermodynamic and kinetic landscapes of the self-assembly process is necessary. Electrostatic forces are important in the self-assembly of a variety of SL structures, but describing the interactions between charged NCs in electrolytic solutions remains a challenge. The interactions between micron-scale charged colloids in electrolytic solutions are well-understood within Derjaguin–Landau–Verwey–Overbeek (DLVO) theory, which describes the interactions as a linear combination of van der Waals attraction, electrostatic repulsion mediated by the electrolytic solution, and steric repulsion.^17^ DLVO theory, however, has limited applicability to nanoscale systems,^18^ especially at high ion concentrations, since the ions in solution are finite in size relative to the NCs and can no longer be considered point charges. As a result, it is challenging to describe and predict the phase behavior of charged NCs in electrolytic solutions as well as the kinetics of their self-assembly into SLs. Therefore, experimental studies of NC SL phase coexistence and the kinetics associated with particular pathways through the associated phase diagram are needed.

Measuring NC systems is challenging due to their fast time and short length scales. Typically, the phase behavior of colloidal systems is measured using optical techniques,^19−24^ but these approaches are ineffective for NCs that fall below the diffraction limit. Synchrotron X-ray scattering is, in principle, an attractive method to address these challenges since it provides nanoscale structural information down to ms time scales.^25−28^ Yet, few *in situ* X-ray scattering studies of NC SL self-assembly exist due to the difficult sample geometries required to follow the full self-assembly process. For electrostatically induced self-assembly, these difficulties include simultaneously processing the initial colloidal suspension, mixing it with reagents, and protecting it from air and humidity, all the while probing a homogenized volume in a thin, X-ray compatible chamber over the full course of the transformation. Furthermore, the few existing studies focus on NCs with organic surface ligands that self-assemble into SLs via spin coating,^25^ solvent evaporation,^25,29−34^ or growth from solution^35−39^ but not on the effect of electrostatic forces on self-assembly. In addition, previous studies primarily measured the kinetics of SL self-assembly under specific conditions and could not also obtain the associated phase diagrams, limiting their ability to directly correlate the kinetics with phase diagram features.

Here, we nevertheless use small-angle X-ray scattering (SAXS) with an apparatus we developed to measure *in situ* and in real time solutions of electrostatically stabilized colloidal Au NCs before, during, and after they are quenched to varying degrees to form SLs. Using a model that we developed for multicomponent solution scattering, we fit the time-dependent SAXS patterns to quantitate the relative amounts of the colloidal and SL phases as well as the crystal structure and crystallinity of the SLs. The SL product and remaining colloidal NC fractions enable mapping of the system phase diagram as a function of colloid concentration and quench depth, which provides insight into the effective range and depth of the interparticle interactions in this system. In addition, the combination of our apparatus, self-assembly protocol, and data analysis techniques enables us to determine how the kinetics of the self-assembly process at different quench depths impact the resulting SL crystallinity and yield and how to alter the protocol for optimal outcomes. Brownian dynamics simulations corroborate the experiments and help to reveal the underlying interparticle interactions and resulting mechanisms of SL growth and annealing. This work presents a generalizable strategy to more completely elucidate the microscopic interactions and self-assembly pathways of a wide range of NC SLs, enabling the design of structures with improved optical, electronic, and mechanical functionalities.

## Results and Discussion

**Figure 1:**
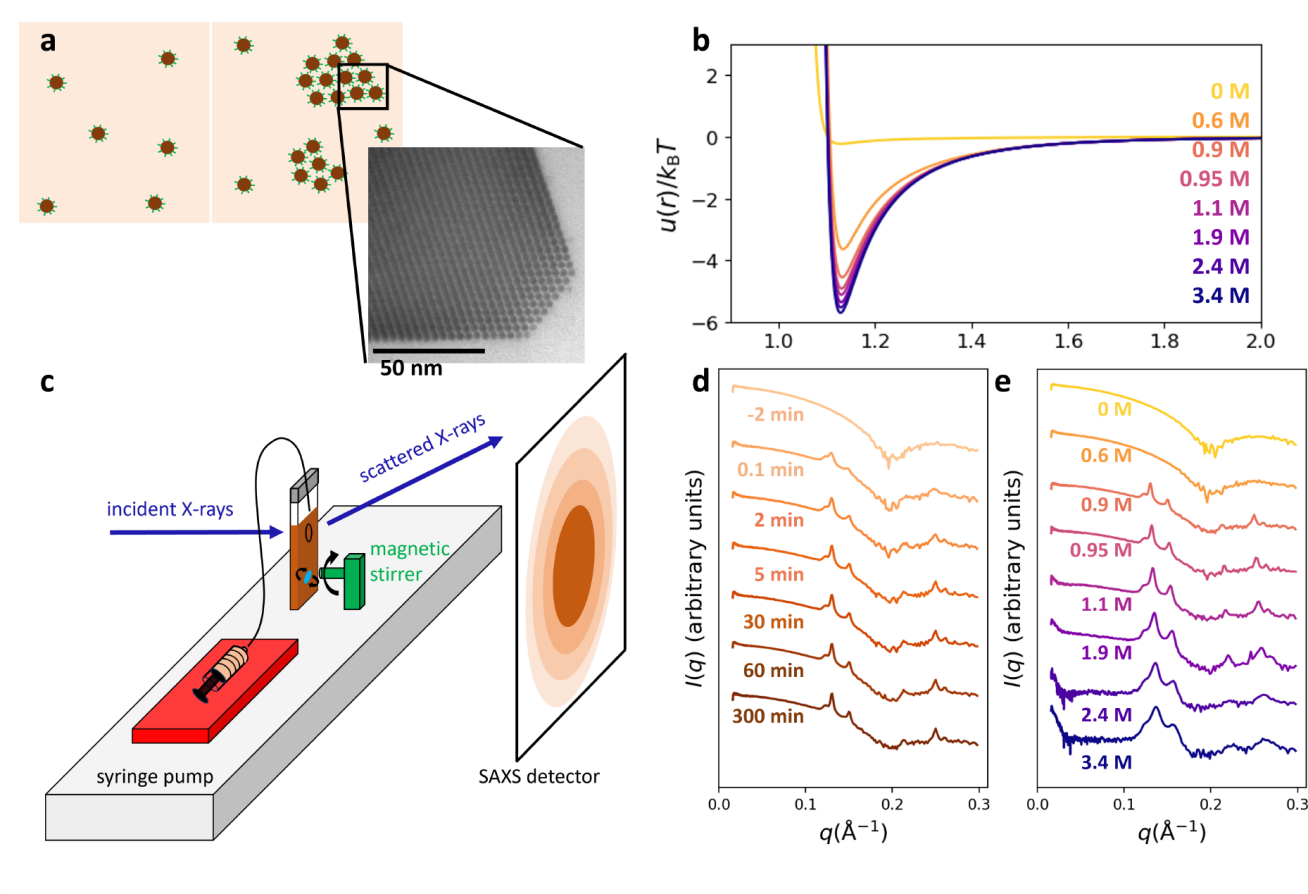
*In situ* monitoring of electrostatic self-assembly of NC SLs. (a) Schematic of colloidal NCs (brown) with thiostannate surface ligands (green lines) in hydrazine (tan) (left) and the coexistence of colloidal NCs with SLs (right). SL domains are not drawn to scale. (inset) TEM image of Au NC SL. Reprinted in part with permission from ref (41). Copyright 2022 The American Association for the Advancement of Science. (b) DLVO interaction potentials vs relevant solution ionic strengths. Here, *u*_0_ is the minimum value of a given curve. (c) Schematic of gas-tight apparatus for measuring X-ray scattering of self-assembly *in situ*. (d) SAXS patterns as a function of time during a typical experiment at a final solution ionic strength of 0.90 M and NC volume fraction of 0.004. (e) SAXS patterns at long times post-quench from *in situ* experiments performed at NC volume fractions ∼0.002 and at different solution ionic strengths.

To determine the effect of electrostatics on the self-assembly of SLs, we study Au NCs with thiostannate (Sn_2_S_6_^4–^) surface ligands colloidally suspended in hydrazine (N_2_H_4_), a polar solvent with a dielectric constant of 52 at room temperature (Figure 1a left). Unlike typical NCs with organic surface ligands, these NCs have charged ligands and NC-NC interactions are thus controlled via electrostatic forces.^40,41^ In order to quench the system to generate a condensed phase (Figure 1a right), we add additional (N_2_H_5_)_4_Sn_2_S_6_ salt solution to the initial ∼50 mg/mL NC suspension, which screens the electrostatic repulsion between NCs and creates overall attractive interactions between NCs. In this study, we systematically varied the final ionic strength of the solution, , from 0.6 mol/L (M) to 3.4 M. Here, *c*_*n*_ is the concentration of ion species *n* in molar, *z*_*n*_ is the valency of ion *n*, and *N* is the number of different ion species in solution. The ionic strength controls the quench depth, i.e., the driving force for self-assembly, which formally is the potential energy difference between a Au NC in the SL and colloidal phases.

Despite its limitations (see Supporting Information), DLVO theory still offers a qualitative understanding of the interparticle interactions as a function of quench depth. Within its framework, the interparticle interactions are determined by linear combinations of the van der Waals attraction, steric repulsion, and electrostatic repulsion between NCs. While the strength of attraction between NCs is set by the van der Waals force, the electrostatics modulate the potential by adding a tunable repulsive force between NCs. For the range of solution ionic strengths studied here, DLVO theory predicts short-range interaction potentials with well depths *u*_0_ ≈ 3–6 *k*_B_*T* (Figure 1b, see Methods for details of the calculations). The thiostannate ligands, which are a key ingredient enabling the self-assembly of ordered SLs, contribute to the high NC surface charge density (i.e., the magnitude of the electrostatic repulsion term) as well as the effective size of the NCs (the steric repulsion term) (see Supporting Information).

To experimentally monitor the self-assembly of Au NC SLs, we developed an apparatus for use with synchrotron small-angle X-ray scattering (SAXS). The gas-tight apparatus consists of a quartz cuvette, with thinned, X-ray transparent windows, connected to a syringe on a syringe pump via tubing inserted into a septum (Figures 1c and S1a,b). A magnetic stirrer rotates a stir bar in the cuvette to homogenize the solution and prevent SLs from sinking to the bottom (Figure S1c). In a typical experiment, the cuvette is initially filled with Au NCs colloidally dispersed in hydrazine, and we quench the system by using the syringe pump to inject over ∼5–12 s a controlled amount of (N_2_H_5_)_4_Sn_2_S_6_ salt dissolved in hydrazine. We collect two-dimensional (2D) SAXS detector images before, during, and after the quench at a rate of one image every 5 s for up to 2 h post-quench. By azimuthally averaging the 2D SAXS detector images, we obtain one-dimensional SAXS patterns, *I*(*q*), that describe the scattered intensity as a function of the scattered X-ray momentum transfer, *q*. The time-dependent series of SAXS patterns provide an ensemble average measure of the evolving phase coexistence of the system over the course of SL self-assembly as well as detailed information about each phase such as the size distribution of the NCs and crystal structure of the SLs. We next describe the observed time- and quench-dependent SAXS patterns and then share how they were fit to a quantitative model to extract the phase diagram of the system and the kinetics of SL formation.

Typical one-dimensional SAXS patterns as a function of time post-quench are shown in Figure 1d. Before the quench, the SAXS patterns show the scattering from the 4.5 nm diameter colloidal NCs. Immediately after quenching the system by bringing the solution ionic strength up to 0.9 M and within the ∼s time resolution of the experiment, *fcc* SL Bragg peaks emerge at the expense of the colloidal phase. The SL Bragg peaks continuously grow and narrow over the entire measurement window. Figure 1e shows ∼1 h post-quench (∼equilibrium) SAXS patterns from 6 experiments at a series of final solution ionic strengths. At a final solution ionic strength of 0.6 M, the system remained purely colloidal. As the quench depth (i.e., solution ionic strength) increases, the widths of the near-equilibrium SL Bragg peaks increase and their peak positions shift to higher *q*, indicating the SLs are smaller, more disordered, and have smaller lattice constants.^42^

**Figure 2:**
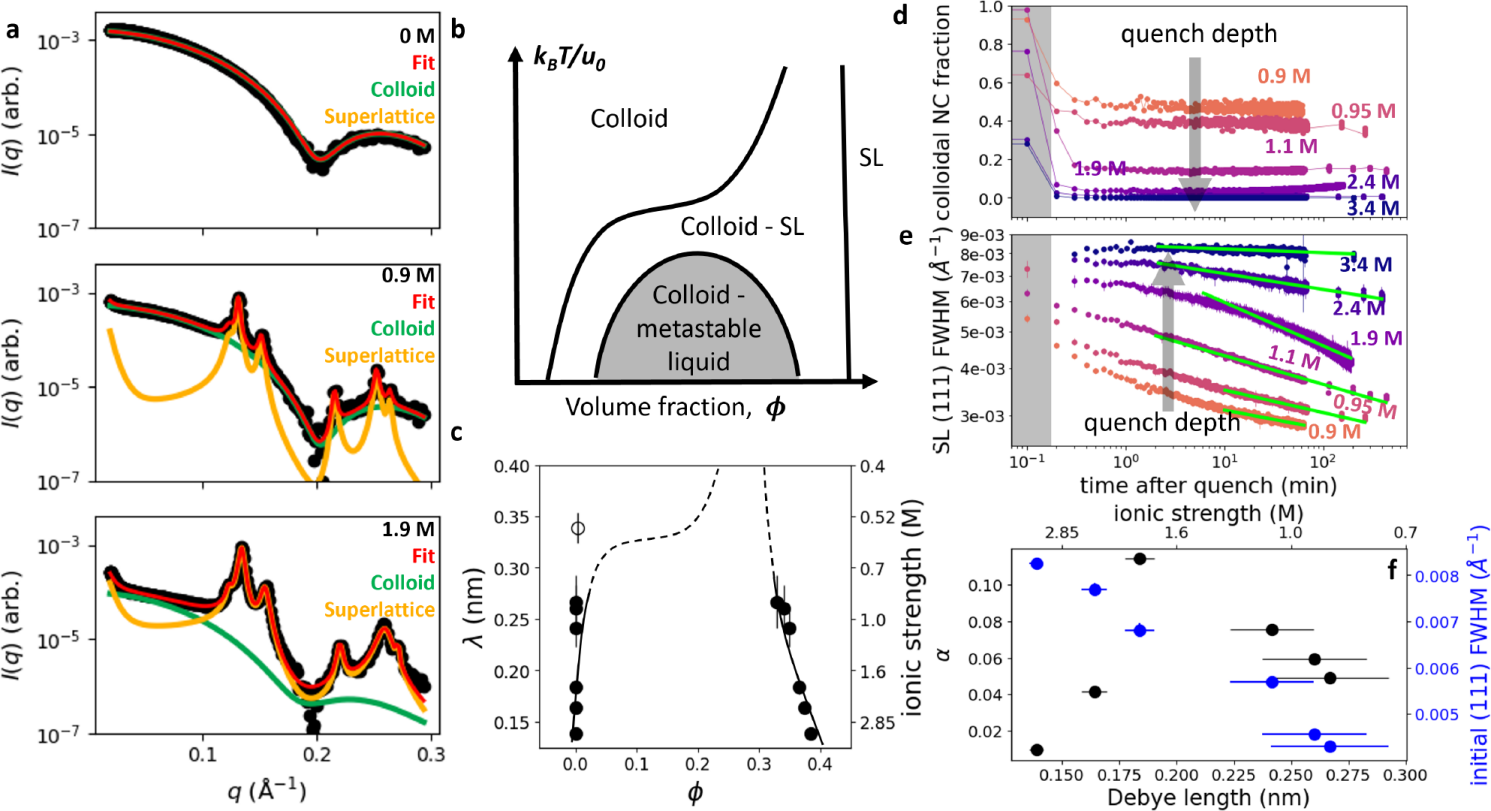
Quantitative analysis of time- and quench-dependent SAXS patterns. (a) Quantitative fits of the model to colloidal NC and SL SAXS patterns at solution ionic strengths of 0, 0.9, and 1.9 M. (b) Schematic phase diagram for spherical particles interacting via short-range potentials. (c) Quantitative phase diagram for electrostatically stabilized Au NCs obtained from experimental observations (black data points). The open circle indicates a phase diagram location where the system is purely colloidal. Vertical error bars indicate the standard deviations of the Debye lengths, λ, of the solutions based on the uncertainty of the volume and concentration of the injected salt solution. Horizontal error bars indicating the standard deviations of the colloidal and SL volume fractions due to the same volume uncertainty and uncertainty from SAXS fitting are smaller than the sizes of the black data points. Black phase boundary curves are sketched as a visual guide based on the location of the black data points. The dashed continuation of these phase boundary curves are sketched to aid comparison to Figure 2b. (d) Fraction of NCs remaining in the colloidal phase as a function of time for a series of quench depths ranging from 0.9 M (orange) to 3.4 M (purple). Gray region indicates the period during which salt injection took place. The same series is treated in panels (e) and (f). (e) fwhm of the SL (111) Bragg peak as a function of time post-quench. Vertical error bars indicate the standard deviations of the fwhm from the SAXS pattern fitting uncertainty. Green lines are power law fits to the fwhm at late times. Gray region same as in (d). (f) fwhm power law exponents, α, as a function of Debye length (black) and the fwhm of SLs upon completion of salt injection (blue). Black vertical error bars indicating the standard deviation in α due to uncertainty from fitting and blue vertical error bars indicating the standard deviation of the fwhm from SAXS pattern fitting uncertainty are both smaller than the size of the data points. Black and blue horizontal error bars are the same as in (c).

In order to extract information from the time-dependent SAXS patterns, we developed and used a model to quantitatively fit them. Specifically, we model the background-subtracted scattered intensity as *I*(*q*) = *I*_colloid_(*q*) + *I*_SL_(*q*), where *I*_colloid_(*q*) is the scattered intensity from dilute, polydisperse hard spheres and *I*_SL_(*q*) is the scattered intensity from finite-sized *fcc* crystals (See Methods and Figures S2 & S3). This model fits the data well at all time points and quench depths (Figure S4), as shown for a selection of final solution ionic strengths in Figure 2a. This fitting scheme allows us to extract the relative amount of NCs in the colloidal and SL phases as well as the degree of crystallinity of the SL phase as a function of time and quench depth.

A cartoon of the expected phase diagram for monodisperse spherical particles with interparticle potentials short in range relative to their diameter is shown in Figure 2b.^43^ The phase diagram consists of binodal (phase boundary) curves that specify the presence and density of each phase as a function of the volume fraction of particles in solution, ϕ, and the effective temperature, *k*_B_*T*/*u*_0_, where *u*_0_ is the depth of the interparticle potential. The ratio *u*_0_/*k*_B_*T* formally defines the quench depth. For example, at high *k*_B_*T*/*u*_0_ and low ϕ, the colloidal phase is the only stable phase. As *k*_B_*T*/*u*_0_ is lowered or ϕ is increased and the left-most binodal is crossed, the solid, or SL, phase becomes thermodynamically stable and exists in equilibrium with the colloidal (gas-like) phase. In addition to the colloidal and SL phases, if an additional binodal is crossed (gray in Figure 2b), then a liquid phase, which consists of densely packed yet fluid colloidal particles with no long-range order, exists as well. Unlike in phase diagrams for typical atomic systems, where the interactions are long-range relative to the size of the atom, the colloid-metastable liquid binodal in the phase diagram in Figure 2b is situated below the colloid-SL binodal. Consequently, the liquid phase is not thermodynamically stable, but previous simulation and experimental work has shown it can exist metastably and even act as a precursor to SL formation.^43−51^ The exact location of the colloid-metastable liquid binodal relative to the colloid-SL binodal depends primarily on the range of the interparticle potential.^52^ Specifically, as the range of interaction decreases, the metastable liquid phase becomes less stable, and the colloid-metastable liquid binodal peaks at lower *k*_B_*T*/*u*_0_.

To determine the thermodynamic landscape of the self-assembly process studied here, we calculated from our experimental data and quantitative fitting the points on the binodal curves that constitute the phase diagram of the Au NC system in Figure 2c. These points specify the volume fraction, or density, of NCs in the colloidal or SL phases, respectively, as a function of quench depth. We use the Debye length 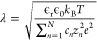 of the solution as a figure of merit for the quench depth on the vertical axis since it combines information on the (varying) ionic strength of the solution and the dielectric constant of the solvent. Here, ϵ_r_ is the solvent dielectric constant, ϵ_0_ is the vacuum permittivity, *e* is the charge of an electron, and *c*_*n*_ and *z*_*n*_ are the same as in the equation for the solution ionic strength. Although this choice does not incorporate the impact of the steric and van der Waals contributions from the NCs into *k*_B_*T*/*u*_0_, these forces should not vary from quench to quench since the same NC stock solution was used for all measurements. To determine the points on the low-density side of the phase diagram in Figure 2c, we compare the scattered X-ray intensity vs *q* of the colloidal phase after equilibrating ∼1 h post-quench to the scattered colloidal intensity prior to the quench. From this ratio, we obtain the fraction of NCs remaining in the colloidal phase as a function of the quench depth. Multiplying this value by the total volume fraction of NCs in the system, we obtain the volume fraction of the colloidal phase at equilibrium with the SL phase for each quench depth. These volume fractions provide the horizontal axis values of the left-hand colloid-SL binodal in Figure 2c. For a given quench depth, the volume fraction of the SL phase determines the points on the high-density colloid-SL binodal, which we obtain from the position of the *fcc* (111) Bragg peak, *q*_111_, using ϕ = 4*V*/*a*^3^, where *V* is the volume of a NC excluding ligands and *a* is the lattice constant of the SL (. The low-density colloid-SL binodal rises steeply with ϕ at low ϕ from ϕ ∼ 0.0 to 0.0009. On the high-density side, the colloid-SL binodal rises less steeply from ϕ ∼ 0.38 to 0.33. In order to resolve the colloid-SL binodals between ϕ ∼ 0.01 and 0.3, larger total NC concentrations than studied here would be required. We did not do so in this work due to greater difficulty in stabilizing the colloidal phase at high NC concentrations. Nevertheless, our observations do constrain the extent of the rise of the low-density binodal at greater ϕ than shown with our data points based on the fact that a final solution ionic strength above ∼0.6 M (λ < 0.34 nm) is needed to generate SLs (open circle in Figure 2c and see in more detail in Figure S5). We sketched the curves between ϕ ∼ 0.01 and ∼0.3 with dashing as an interpolation between the solid curves dictated by experimental data; the dashed curves are not meant to be quantitative. At all time points and quench conditions studied here, we did not observe a liquid phase in the SAXS patterns, and as a result, our measured phase diagram consists solely of colloid-SL binodals.

To characterize the time evolution of the colloidal and SL phases, we also extracted the kinetics of each phase. The kinetics of the colloidal phase, which are anticorrelated with those of the SL phase (not shown), in Figure 2d are extracted similarly to the method described above to determine the equilibrium fraction of colloidal NCs for the phase diagram in Figure 2c. We find that the fraction of NCs remaining in the colloidal phase decreases monotonically with the quench depth. While the fraction of NCs remaining in the colloidal phase as a function of time in any given quench also decreases monotonically, the decrease is very small in magnitude following the salt injection period, shaded in gray in Figure 2d. This finding suggests that the colloid fractions following the initial quench approach the thermodynamically expected values.

In Figure 2e, we determined the full width at half-maximum (fwhm) of the *fcc* (111) SL Bragg peak as a function of time and quench depth. This quantity encodes both the coherence length of the SL, i.e., the typical length scale of a crystalline domain, and the degree of crystallinity of those domains. We focused our analysis on the SL (111) peak since the limited *q*-resolution and signal-to-noise of the higher-order Bragg peaks limited the reliability of more involved analysis methods such as Williamson-Hall and Debye–Waller analyses. The SL (111) fwhm immediately after the injection increase monotonically with the quench depth (Figure 2f blue points) and decrease monotonically with time at all studied quench depths (orange to purple, respectively, from 0.9 to 3.4 M in Figure 2e). We fit the late-time behavior to a power law, fwhm ≈ *t*^–α^ (Figure 2e green lines, see Supporting Information), and find that α depends nonmonotonically on the quench depth (Figure 2f black points). At the shallowest SL-producing quench to λ = 0.267 nm (0.9 M), α = 0.049 ± 0.001. As the quench depth increases, α increases and reaches a maximum of 0.110 ± 0.001 at λ = 0.184 nm (1.9 M). As the quench depth continues to increase, α decreases and has a value of 0.010 ± 0.001 at the deepest quench depth at λ = 0.139 nm (3.4 M).

**Figure 3:**
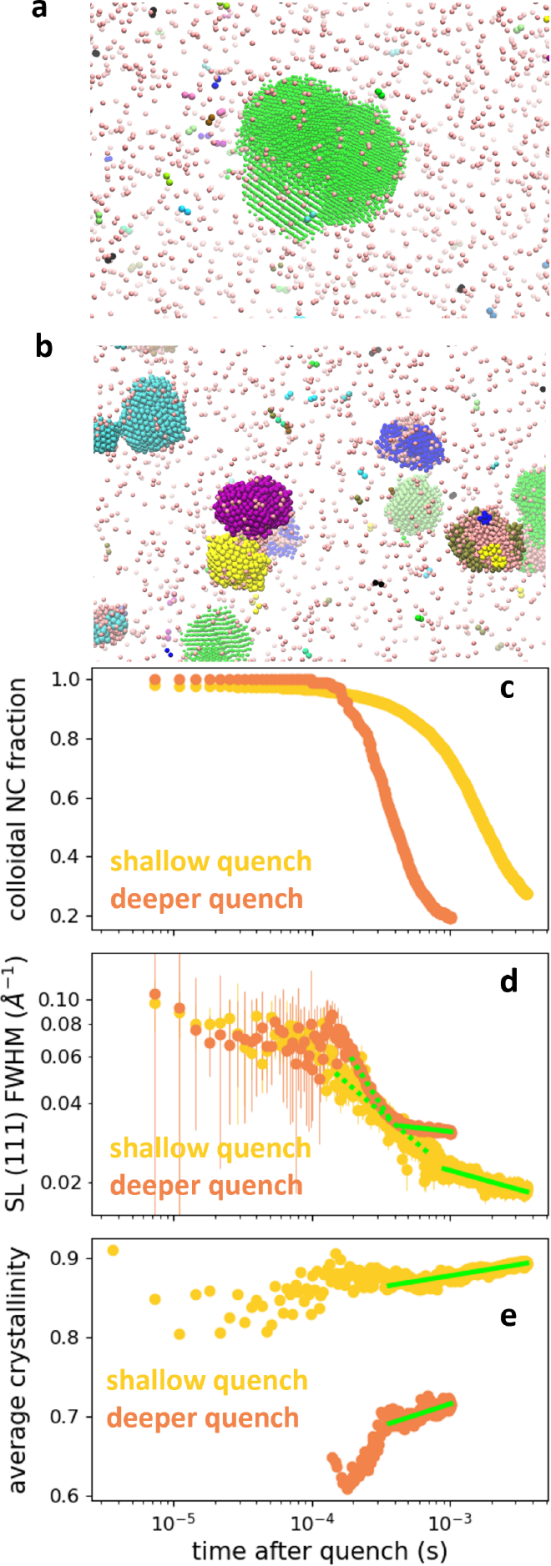
Simulations of SL self-assembly. (a) Snapshot of the shallow quench simulation. The different colors correspond to different crystalline clusters (see Methods for cluster determination), with colloidal NCs shown in light pink. The visualized NC size is reduced by 70% from 4.5 nm to aid visualization. (b) Snapshot of the deeper quench simulation at same scale as in (a). Colors and NC sizes determined as in (a). (c) Simulated colloidal NC fractions vs time for shallow quench (yellow) and deeper quench (orange). (d) Simulated SL *fcc* (111) Bragg peak fwhm vs time for shallow quench (yellow) and deeper quench (orange). Vertical error bars indicate standard deviation in SL (111) fwhm due to uncertainty from fitting procedure. Power law fits to early times are shown in dotted green and to later times are shown in solid green. (e) Average crystallinity of particles in the condensed phase vs time for both quenches. Colors are the same as in (c) and (d). Power law fits to late times are shown in solid green.

To determine the relationships among the SL (111) fwhm, the colloidal NC fractions, and the underlying self-assembly mechanisms, we simulated the self-assembly process at two different quench depths. The simulations were performed in the NVT ensemble, representing the NCs as spherical particles interacting through a coarse-grained, short-range attractive Morse potential. Starting from a homogeneous phase, we quenched the NCs to two different depths of the interaction potential (*u*_0_/*k*_B_*T*) and studied the dynamics of the growth of dense and ordered clusters (see Methods and Supporting Information for details of the simulations). Snapshots of the simulations are shown in Figure 3a,b. For the shallow quench simulation, we induced nucleation with a spherical *fcc* seed, while in the deeper quench, nucleation of spherical liquid droplets occurred spontaneously. SLs nucleated from within the liquid droplets and subsequently grew and annealed. Every 20 Brownian time units, we calculated the expected X-ray scattering patterns of the system and fit them using our model (Methods and Figure S6). The simulated colloidal NC fractions for both quenches in Figure 3c decrease monotonically, indicating that NCs transfer from the colloidal phase to the SL phase as the SLs grow. Figure 3d shows the simulated *fcc* (111) Bragg peak fwhm for the two quench depths. We uncover two kinetic regimes that we classify with power laws. As indicated by the dotted green lines, the ∼sub-ms kinetics follow a *t*^–α^ power law with α = 0.435 ± 0.013 and α = 0.447 ± 0.008 for the shallow and deeper quenches, respectively. We do not observe this regime in the experiments due to the limited time resolution associated with finite-time injection and the SAXS detector acquisition rate. At longer ∼ms times, the power laws of the simulated kinetics become more similar to the experimental values obtained on ∼s to hour time scales with α = 0.116 ± 0.002 for the shallow quench simulation and α = 0.063 ± 0.003 for the deeper quench simulation, each indicated with solid green lines in Figure 3d.

With access to the real space positions of every simulated NC, we calculate the ensemble-averaged crystallinity of NCs in the SL phase by tracking for each NC a Steinhardt-Nelson order parameter^53^ for orientations of bonds with neighboring NCs (see Methods). Unlike the fwhm of the Bragg peaks, the average crystallinity decouples the finite size of the SLs from the degree of order of the SL domains. The average crystallinities of the SLs in the two simulations are shown in Figure 3e. We find that the SLs in the deeper quench simulation have lower average crystallinity than the SL in the shallow quench simulation. As indicated by the green lines, the late time average crystallinity kinetics also follow a power law, with α = 0.014 ± 0.001 for the shallow quench and α = 0.035 ± 0.002 for the deeper quench.

Having described our observations, SAXS analysis strategies, and corroborating simulations, we turn toward a discussion of our findings and their implications for NC SL self-assembly. We begin by commenting on the features of the phase diagram extracted from the experiments as well as the insight the phase diagram provides into the effective range and strength of the NC-NC interparticle interactions in this system. We discuss these insights in relation to the predictions from DLVO theory. Next, we summarize the different kinetic regimes (nucleation/growth, and coarsening) of SL self-assembly and how they impact the resulting SLs. Finally, by comparing how the early- and late-time kinetics vary as a function of the quench depth in experiment and in simulation, we propose kinetic design principles for optimal SL self-assembly in a finite time.

With our measurement and analysis protocol, we can directly and quantitatively reveal a substantial portion of the phase diagram for the electrostatically stabilized Au NCs studied in this work. The phase diagram is consistent with the expectation that as the quench depth increases, the equilibrium volume fraction of the colloidal phase decreases as more colloidal NCs are incorporated into SLs (Figures 2c and S5). In addition, the volume fraction of the SL phase increases as the quench depth increases. This trend could be due to the depth (*u*_0_) of the interparticle potentials becoming greater at deeper quenches or due to NC size-selectivity during self-assembly, i.e., larger NCs condensing before smaller ones due to stronger van der Waals attraction. We propose that NC size-selectivity is the most likely reason based on estimation using a statistical analysis of NC diameters, nearest neighbor distances between NCs in the SL phase at different quench depths, and scalings of van der Waals attraction vs NC diameter (see Figure S7 and associated text). One additional limitation of obtaining the phase diagram with the methods described in this work is that the systems ∼1–2 h post-quench at different quench depths may not all be similarly close to equilibrium. Although the colloidal NC fractions following the initial quench approach the thermodynamically expected values (Figure 2d), if the systems measured were not exactly at equilibrium, the true low-density colloid-SL binodals would be located at slightly smaller ϕ than we obtained. While it is difficult to know exactly at which densities the equilibrium states will be, we estimate that the colloidal NC fractions at equilibrium are within a few percent of the corresponding fractions ∼1 h post quench (see Supporting Information). At deeper quenches, the SL (111) fwhm increase, indicating that the SLs are further away from their equilibrium structures. As a result, the high-density colloid-SL binodal we extracted may very slightly underestimate the true equilibrium SL density.

Despite the small uncertainty in the precise locations of the phase diagram binodals, we can use the phase diagram and evolving phase coexistence to obtain insight into the nature of the interparticle interactions governing the self-assembly process. For example, we find no presence of a liquid phase in any of the SAXS patterns obtained after equilibration (or at any time) at any quench depth. The absence of a thermodynamically stable liquid phase implies that these NCs interact via short-range attractive potentials. Even though we do not experimentally observe a metastable liquid phase, the phase diagram for this system is consistent with phase diagrams of particles with short-range interactions because it consists solely of regions where either the colloidal phase is the only stable phase or the colloid and SL coexist. The phase diagram is inconsistent with those of hard spheres or particles interacting via long-range interactions because SLs form at volume fractions lower than ϕ = 0.49, the freezing density of hard spheres, and there is no region of colloid-liquid coexistence at shallow quenches as there would be for particles with long-range attractive interactions. One reason we may not observe the metastable liquid phase is that it may convert entirely into SLs beneath the time resolution of the measurement (∼5 s). Indeed, in the deeper quench simulation, SLs nucleate and grow from within liquid droplets on average in <200 μs (Figure S8a). Another possibility is that the range of the interparticle potential is sufficiently short that it suppresses the colloid-metastable liquid binodal sketched in Figure 2b far enough below the colloid-SL binodal that it is experimentally inaccessible under our quenching conditions. In other words, our quenches may place the system only in the colloid-SL coexistence region between the colloid-SL and colloid-metastable liquid binodals in Figure 2b (see Figure S8b). Our previous work on the self-assembly of SLs from these NCs showed the formation of dense agglomerations of NCs after similar amounts of salt solution as used in this study were added to the initial colloidal NC suspensions in combination with a acetonitrile antisolvent.^41^ The additional acetonitrile may have quenched the solution to a low enough λ to access the metastable liquid binodal. While that more aggressive quench protocol followed its own specific kinetic trajectory, it is also possible that the liquid state could be kinetically forbidden using the less aggressive protocols in this present work at even higher ionic strengths, if quenches too shallow to cross the metastable liquid-colloid binodal were already to lead to kinetic arrest.

**Figure 4:**
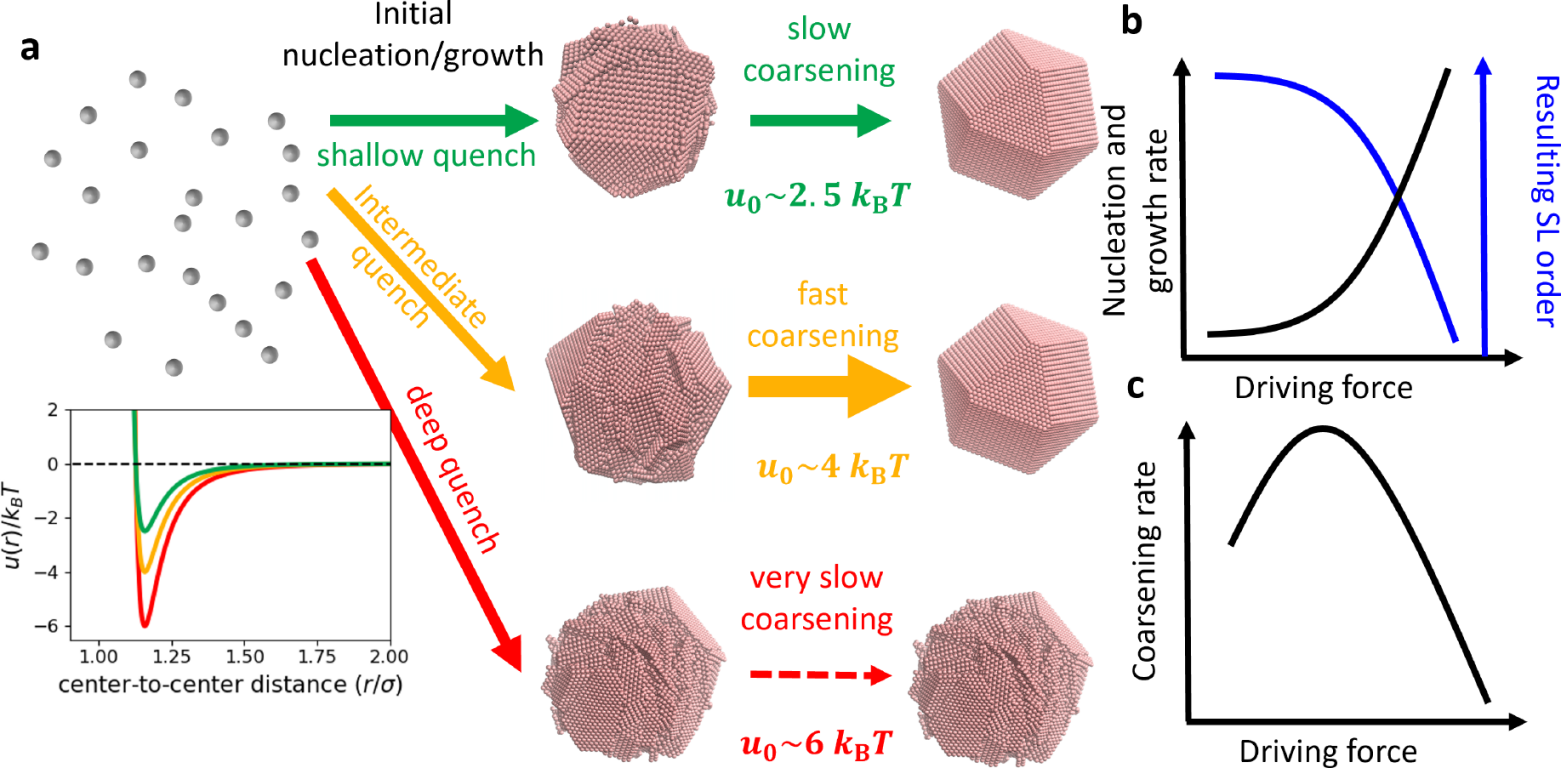
SL formation mechanisms and time scales. (a) Top: at shallow quench depths, highly ordered SLs nucleate and grow from solution at time scales <1 s. These SLs grow and anneal defects slowly over the course of minutes to hours. Middle: at intermediate quench depths, slightly more disordered SLs nucleate initially, and over longer time scales the SLs grow and anneal away the disorder at a faster rate than at shallow quenches. Bottom: in deep quenches, disordered SLs form and are unable to anneal away defects due to kinetic trapping. Inset: interparticle potentials for shallow (green), intermediate (gold), and deep (red) quenches. (b) Sketch of SL nucleation and growth rate and order of the resulting SLs as a function of the driving force for self-assembly. (c) Sketch of rate at which SLs coarsen as a function of the driving force for self-assembly.

In principle, knowledge of the exact location of the colloid-metastable liquid binodal relative to the colloid-SL binodal enables determination of the effective range of the interparticle potential. Since we did not observe any liquid phase and therefore could not map out a colloid-metastable liquid binodal, we are unable to precisely specify the range of the interparticle potential. Nevertheless, since we do not observe a thermodynamically stable liquid phase, we can infer from the Noro-Frenkel law of corresponding states^52^ that the center-to-center range of the interparticle potential must be <1.2 σ_eff_, where σ_eff_ is the effective size of a NC, including its ligand shell. Combined with the measured nearest neighbor distances between NCs in the SL phase (Figure S7b), we estimate the effective size of the NCs to be ∼5.6–5.9 nm, resulting in an effective center-to-center range of interparticle interactions of ∼6.7–7.1 nm, i.e., no greater than 1.2 σ_eff_. Interestingly, as a result, the DLVO theory predictions of the range of interactions for this system under the quench conditions studied here (Figure 1b) were reasonable. DLVO theory, however, overestimates the depth (*u*_0_) of the interparticle potentials. We base this conclusion on the following reasoning. First, DLVO predicts a well depth of ∼3*k*_B_*T* for a solution ionic strength of 0.6 M (Figure 1b). At 0.6 M, however, the system remained purely colloidal (Figure 1e orange curve), which could only occur if the well depth were less than ∼2*k*_B_*T*.^54^ In addition, well depths of 2.5 and 2.8 *k*_B_*T* used in the simulations, respectively, resulted in colloidal NC fractions of ∼0.27 and 0.19 at the end of the simulated trajectories. These fractions are similar in magnitude to those obtained from experiment ∼1 h after a shallow to intermediate quench. This similarity indicates that values of *u*_0_ smaller than those predicted by DLVO theory produce colloidal NC fractions that are consistent with the experimental results (see Supporting Information). These findings suggest that while DLVO theory provides qualitatively accurate predictions for the range of the interparticle potential, it fails quantitatively in its prediction of the depths of the interparticle potentials for this charged NC system. Although DLVO predictions may agree with the inferred potential depths of the deeper experimental quenches (perhaps as deep as 6 *k*_B_*T*), based on our simulations and additional computational work,^45,54^ we expect the potential depths of shallower quenches to be closer to 2.5 *k*_B_*T*, which explicitly disagrees with DLVO. The inferred interparticle potentials between NCs following shallow, intermediate, and deep quenches in experiments are shown in Figures 4a and S9. *In situ* experimental approaches combined with quantitative analysis and simulation tools as presented in this work thus provide a means by which to determine the nature of the underlying interactions.

While the long-time behavior of the system post-quench provides insight into the nature of the interparticle interactions and general thermodynamic landscape of the system as a function of λ and ϕ, the full time evolution of the self-assembly process reveals two distinct kinetic regimes. On sub-millisecond time scales in the simulations, the SL (111) fwhm decrease as power laws with power law exponents ∼0.45 corresponding to the initial nucleation and growth of the SLs from the colloidal phase, which we do not resolve experimentally. On ∼ms time scales in the simulations and on min-hr time scales in the experiments, the SL (111) fwhm decrease at much smaller power law rates. These slower kinetics correspond to the coarsening stage of the self-assembly process. During this stage, the SL (111) fwhm in the experiments and simulations decrease due to the annealing of defects. While coherent X-ray scattering experiments can be used to more explicitly specify the nature of the disorder being annealed,^55^ this observation could be due to either the average crystallinity increasing within each SL domain or the annealing of grain boundaries separating distinct SL grains within a single polycrystalline SL. The simulated SL (111) fwhm additionally decrease due to SL growth via incorporation of NCs from the colloidal phase. These time scales indicate that the SLs in the simulation leave the initial nucleation and growth regime and enter the coarsening regime within a few ms postquench. This finding also suggests that the SLs observed in the experiments have already entered the coarsening regime during the injection period.

In order to further determine the impact of the two kinetic regimes on the self-assembly process, we discuss the kinetic trends as a function of the quench depth. The trends in the initial SL (111) fwhm and the late-time power law exponents as a function of the quench depth in the experiments (Figure 2f) reveal that at shallow quench depths, the initially formed SLs are very ordered and only slowly coarsen over the course of minutes to hours (Figure 4a top). As the quench depth increases, the SLs are initially more disordered but are able to anneal these defects faster than is possible at shallow quenches (Figure 4a middle). As the quench depth increases further, the SLs become much more disordered and can no longer anneal away defects due to kinetic trapping, even though the thermodynamic driving force for self-assembly is even stronger (Figure 4a bottom). The simulations show qualitatively similar trends: after the initial nucleation and growth period, the SLs in the simulated deeper quench have larger fwhm (Figure 3d) and lower crystallinity (Figure 3e) than in the shallow quench. Although the simulations cannot access the same long time scales as in the experiments, the SLs in the simulation already enter the coarsening regime within the simulated time frame (∼a few ms). During the coarsening stage in the simulations, the SLs continue to grow via the incorporation of colloidal NCs and to anneal defects. As a result, the SL (111) fwhm in the shallow quench simulation decrease with a larger power law exponent (α = 0.116) than those in the deeper quench simulation (α = 0.063) primarily due to faster growth. This behavior is distinct from the behavior on min-hour time scales in experiment since the colloidal NC fractions have plateaued by then, and the decrease in the SL (111) fwhm is primarily due to defect annealing. In fact, during the coarsening stage, the simulated deeper quenched SLs anneal various defects and increase their average crystallinity at a faster power law rate (α = 0.035) than the SL in the simulated shallow quench (α = 0.014). While polydispersity could also play a role in this latter outcome,^56,57^ this trend supports the idea that the SL (111) fwhm at intermediate quenches in the experiment decrease at faster rates than those at shallow quenches in experiment due to an increased ability to anneal remaining defects and increase their crystallinity. Although not explicitly simulated here, even deeper quenches would likely lead to kinetic arrest,^45,54^ which qualitatively agrees with the trend observed experimentally in Figure 2e,f because it would corroborate the nonmonotonic behavior in the coarsening rate vs quench depth.

By combining the kinetic trends as a function of quench depth in the experiment and simulation, we propose emergent design principles for NC SL self-assembly, even beyond the electrostatically stabilized system studied here. Typically, the nucleation and growth rate increases exponentially with the quench depth or driving force for self-assembly (Figure 4b black curve). The conventional wisdom is that larger driving forces lead to faster kinetics, which ultimately results in SLs that are less ordered than SLs assembled more slowly (Figure 4b blue curve). Immediately post-quench, the fwhm of the SLs as a function of quench depth in the experiments (Figure 2e,f) and in the simulations (Figure 3d) support this trend. Surprisingly, the coarsening rate for this system (Figure 2f) does not follow this trend and instead depends nonmonotonically on the driving force for self-assembly (Figure 4c). This finding suggests a *kinetic* strategy to improve SL self-assembly. Specifically, using a small driving force to nucleate and grow the initial SLs and then increasing the driving force to better promote *in situ* coarsening should facilitate the self-assembly of more highly ordered SLs within a finite amount of time.

## Conclusion

In summary, we presented an improved method by which to measure the self-assembly of NC SLs *in situ* and in real time using synchrotron X-ray scattering. We developed a quantitative model to fit time-dependent SAXS patterns of NCs in the colloidal and SL phases, which enabled us to extract the phase diagram and kinetics of the transformation under different conditions. By combining the insights from simulation with experiment, we have shown the ability to elucidate the effective range and depth of interactions between charged NCs in electrolytic solutions, which are consistent with short-ranged potentials. We also found the SL self-assembly kinetics have two regimes (nucleation/growth and coarsening) and that the coarsening kinetics depend nonmonotonically on the driving force for self-assembly. Consequently, we propose kinetic strategies to promote ordered SL self-assembly by increasing the driving force for self-assembly as a function of time.

Identifying this proposed design protocol was only possible thanks to the powerful combination of *in situ* measurement, quantitative analysis, and simulation used in this work, which unveiled the equilibrium properties and nonequilibrium effects that underlie NC SL self-assembly. In particular, this approach should be able to reveal the impact of a metastable liquid phase on SL formation and protein crystallization in systems in which the metastable liquid either has a longer lifetime or is stable at lower Debye lengths. More generally, a similar approach could be used to design protocols for other related systems, such as in protein crystallization^48,58^ and the formation of other hierarchical materials such as metal-^59−61^ and covalent-organic frameworks,^62−64^ by determining first how to tune the self-assembly driving force from an understanding of the interparticle/intermolecular interactions and resulting thermodynamic landscape and, second, how to apply the driving force based on the kinetics. The approach should also be generalizable to elucidate the microscopic pathways and design principles of a variety of nanoscale self-assembly phenomena, for example, in enhancing the complexity of DNA-based nanomachines^65^ and optimizing nanostructures for drug delivery.^66^

## Methods

### DLVO Calculations

The DLVO interaction potentials in Figure 1b are the sum of three terms: *u*(*r*) = *u*_es_(*r*) + *u*_st_(*r*) + *u*_vdW_(*r*), where *u*_es_(*r*) describes the contribution from the electrostatic interaction between two charged NCs, *u*_st_(*r*) describes the contribution from the steric overlap of the NCs at small *r*, *u*_vdW_(*r*) describes the van der Waals attraction between the NCs, and *r* is the center-to-center distance between two NCs. The electrostatic contribution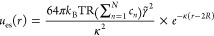, , where *R* is the radius of the NCs, *c*_*n*_ is the concentration of ion *n*, *N* is the number of different ion species in solution, κ = λ^–1^, and  where *z* is the valency of the NC surface ligand, *e* is the charge of an electron, and ψ is the NC surface potential. For the calculations in this work, values of ψ such that γ̃ = 1 were used. The van der Waals contribution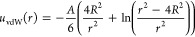 , where *A* = 1.6 eV is the Hamaker constant of gold (see Supporting Information).^67^ For *u*_st_(*r*), we use an exponential with a steep cutoff at 0.5 nm (roughly the size of a single ligand molecule): *u*_st_(*r*) = *ae*^–*b*(*r*–σ)^, where *a* = 5 × 10^4^*k*_B_*T* and *b* = 5.4 × 10^–11^ nm^–1^.

### In Situ SAXS Experiments

All SAXS data were collected at the Stanford Synchrotron Radiation Lightsource (SSRL) at beamline 1–5 with a photon energy of 15 keV and beam size of 600 × 600 μm (see Figure S1 for further information). Stock solutions of 4.5 nm Au NCs with (N_2_H_5_)_4_Sn_2_S_6_ ligands in hydrazine and 0.5 M (N_2_H_5_)_4_Sn_2_S_6_ salt in hydrazine were prepared following a procedure previously outlined.^40,41^ In a nitrogen-filled glovebox, 400–500 μL of a 50 mg/mL (ϕ ∼ 0.0026) solution of 4.5 nm Au NCs with (N_2_H_5_)_4_Sn_2_S_6_ ligands in hydrazine was loaded into a 2 mm path length quartz cuvette with custom 200 μm thick windows. A small stir bar was placed into the cuvette in the plane of the cuvette, and the cuvette was then sealed using a rubber septum and parafilm. A syringe preloaded with a solution of 0.5 M (N_2_H_5_)_4_Sn_2_S_6_ in hydrazine was attached to the cuvette via Teflon tubing through the septum. The tubing-septum interface was sealed with epoxy. The gas-tight apparatus was carefully moved into the beam path, and the syringe was placed onto a New Era syringe pump (model NE-1000). X-ray scattering data were collected continuously while the solution was stirred using a magnetic stirrer from Ultrafast Systems. All X-ray scattering patterns were collected by using 1 s exposures at a rate of one pattern every 5 s. For each *in situ* experiment, after about 5 min of data acquisition, the excess salt in hydrazine solution was injected using the syringe pump at a rate of 847.6 μL/s. The injection took ∼5–12 s depending on how much salt was added. The total volume fraction of NCs in solution post-injection varied from ∼0.0017 to ∼0.0024 depending on the volume of the salt solution that was injected. The apparatus was kept at room temperature (see Supporting Information for additional temperature considerations). Data were continuously acquired after injection for up to 2 h. SAXS patterns of cuvettes filled with hydrazine and varying amounts of (N_2_H_5_)_4_Sn_2_S_6_ salt were taken for background subtraction (see Figure S2). Because the scattering from NCs depends only on the magnitude of the scattered X-ray momentum transfer, |***q***| = *q*, and because the scattering from SLs results from many SLs at different orientations with respect to the X-ray beam, we azimuthally average the 2D SAXS detector images without loss of information to obtain one-dimensional SAXS patterns, *I*(*q*), that describe the scattered intensity as a function of *q*.

### Modeling of SAXS Patterns

We model the background-subtracted scattered intensity as *I*(*q*) = *I*_colloid_(*q*) + *I*_SL_(*q*), where *I*_colloid_(*q*) is the scattered intensity from colloidal NCs and *I*_SL_(*q*) is the scattered intensity from finite-sized *fcc* SLs. For *I*_colloid_(*q*), we use the form factor for dilute, polydisperse, hard spheres with a Gaussian size distribution. We calculate the form factor using xrsdkit (https://github.com/scattering-central/xrsdkit). For the SL term, we multiply the form factor by the structure factor for a finite-sized *fcc* SL. We model the SL structure factor as the sum of a set of Lorentzian line shapes each centered on a respective Bragg peak of an *fcc* lattice and an additional *q*^–4^ term. We fit our model to the experimental SAXS patterns, *I*(*q*), to obtain the SL Bragg peak positions and fwhm and the relative amounts of NCs in the colloidal and SL phases. For more information and justification of this model to describe the scattering from finite-sized SLs, see Supporting Information and Figure S3 and the associated text.

### Simulations of Self-Assembly

Simulations of SL growth and annealing were performed with an underdamped Langevin dynamics in the NVT ensemble in a cubic periodic box using the LAMMPS software.^68^ NCs were represented as 10976 spherical particles with pairwise volume exclusion interactions given by a Weeks–Chandler–Andersen (WCA) potential.^69^ Additionally, NCs interact also via a pairwise attractive short-range Morse potential. At a given temperature *T*, the diffusive time scale for NCs is given by τ = *γσ*^2^/*k*_B_*T* where σ is the NC diameter, γ is the friction coefficient in Langevin dynamics, and *k*_B_ is Boltzmann constant. To compare to experimental time scales, we assume that the NCs follow Stokes’ law of diffusion, with friction coefficient relating to the solvent viscosity η as γ = 3*πησ*. We then use σ = 4.5 nm, η = 0.876 × 10^–3^ Pa-s, and *T* = 300 K to obtain τ = 0.18 μs. NCs were initially equilibrated in the gas phase before being adiabatically quenched to *u*_0_ = 2.5*k*_B_*T* and *u*_0_ = 2.8*k*_B_*T* for the shallow and deeper quench, respectively. In the case of the shallow quench, we used a spherical defect-free *fcc* crystal of size 200 NCs as a seed to start crystal growth and annealing. For more details on the simulations, see the Supporting Information.

### Crystallinity Calculation

We tracked crystalline order during the simulated self-assembly trajectories by computing for each NC its local bond-orientational order, ψ_6_^(*i*)^, using Steinhard-Nelson order parameters.^53^ At each time frame, we define a NC’s nearest neighbors as all other NCs within a center-to-center cutoff distance of 1.5σ. A NC was defined to be locally crystalline if either its ψ_6_^(*i*)^ parameter was above a cutoff of 0.7^53^ or if its neighbor was locally crystalline. All locally crystalline particles are then classified into clusters of direct or indirect neighbors. All reported results about the crystallinity of NCs in the SL phase are computed only over NCs in these dense locally-ordered clusters containing at least 100 NCs. For more details on the local bond-orientational order calculations, see Supporting Information.

### Simulated Scattering Patterns

We calculated the structure factor, *S*(*q*), of the particles in the simulations every 20τ time units using the formula , where **r**_*n*_ is the location of particle *n* and *N* = 10976. We then average over shells of constant *q* = |**q**| to obtain *S*(*q*). We fit our model for the SL structure factor to the simulated *S*(*q*) to extract the fwhm and Bragg peak positions (see Figure S6).

## Supporting Information Available

Experimental details, modeling of SAXS patterns, effects of polydispersity on the phase diagram, SL (111) fwhm power law exponents, simulation details, defect annealing in simulations (PDF)

## Notes

A preprint version of this manuscript can be found in the arXiv preprint server.^70^
