# Supplementary material for: In Situ X-ray Scattering Reveals Coarsening Rates of Superlattices Self-Assembled from Electrostatically Stabilized Metal Nanocrystals Depend Nonmonotonically on Driving Force
Source: ACS Nano. 2024 Feb 6;18(7):5778–89. doi: 10.1021/acsnano.3c12186 (PMC10883038; doi:10.1021/acsnano.3c12186)
Supplement: Supplementary file 1 — nn3c12186_si_001.pdf [file nn3c12186_si_001.pdf]

Supporting Information for In situ X-ray scattering reveals coarsening rates of superlattices self-assembled from electrostatically stabilized metal nanocrystals depend non-monotonically on driving force

Christian P. N. Tanner,[†] James K. Utterback,[†,‡] Joshua Portner,[¶] Igor Coropceanu,[¶] Avishek Das,[†,§] Christopher J. Tassone,[‖] Samuel W. Teitelbaum,[⊥] David T. Limmer,[†,#,@,△] Dmitri V. Talapin,[¶,∇] and Naomi S. Ginsberg[*,†,††,‡‡,¶¶,△,§§]

†*Department of Chemistry, University of California, Berkeley, CA 94720, USA*

‡*Present Address: Sorbonne Université, CNRS, Institut des NanoSciences de Paris, INSP, 75005 Paris, France*

¶*Department of Chemistry, James Franck Institute, and Pritzker School of Molecular Engineering, University of Chicago, Chicago, IL 60637, USA*

§*Present Address: AMOLF, Science Park 104, 1098 XG, Amsterdam, The Netherlands*

‖*Stanford Synchrotron Radiation Lightsource, SLAC National Accelerator Laboratory, Menlo Park, CA 94025, USA*

⊥*Department of Physics, Arizona State University, Tempe, AZ 85287, USA*

#*Chemical Sciences Division, Lawrence Berkeley National Laboratory, Berkeley, CA 94720, USA*

@*Materials Sciences Division, Lawrence Berkeley National Laboratory, Berkeley, CA 94720, USA*

△*Kavli Energy NanoSciences Institute, University of California, Berkeley, CA 94720, USA*

∇*Center for Nanoscale Materials, Argonne National Laboratory, Argonne, IL 60517, USA*

††*Department of Physics, University of California, Berkeley, CA 94720, USA*

‡‡*Molecular Biophysics and Integrated Bioimaging Division, Lawrence Berkeley National Laboratory, Berkeley, CA 94720, USA*

¶¶*Materials Sciences and Chemical Sciences Division, Lawrence Berkeley National Laboratory, Berkeley, CA 94720, USA*

§§*STROBE, NSF Science & Technology Center, Berkeley, CA 94720, USA*

E-mail: nsginsberg@berkeley.edu

Nanocrystal surface chemistry. The nanocrystal solvation shell is currently under investigation because its characteristics are more complex than a typical double layer.[1–3] The ζ-potential for the type of NCs used in this study was previously measured[4] to be $\sim$ -0.4 V. We do not expect these Au NCs to be faceted due to their small size. With the addition of excess $(N_2H_5)Sn_2S_6$ salt, we do not expect the surface charge to change but we do expect the concentration of ions near the NC surface to increase based on simulation work.[5] Self-assembly of NCs into ordered SLs requires the addition of a salt containing multivalent ions.[6]

Limitations of DLVO theory. DLVO theory treats ions in solution as point particles. This assumption breaks down for NCs whose diameters are comparable in size to the ions themselves,[3] which is the case for the Au NCs (4.5 nm) and $Sn_2S_6^{4-}$ ions ($\sim$0.7 nm) in this work. The sizes of the ions are also comparable to and even larger than the calculated Debye lengths ($\sim$0.14-0.27 nm). In addition, the Hamaker constant used in the DLVO calculations in this work is taken from measurements between (flat) gold surfaces in water[7] since accurate measurements for NCs are lacking.

Temperature stability of the apparatus. The *in situ* apparatus is not thermally stabilized, however, we do not expect the temperature of the apparatus to change by more than a few degrees C (or $\sim$0.01 k_BT, which is negligible on the scales of the well depths) at most due to fluctuations in room temperature and absorption of X-rays based on the X-ray flux, beam size, and the specific heat of the sample.

Modeling of SAXS patterns. We model the background-subtracted scattered intensity as $I(q) = I_{\mathrm{colloid}}(q) + I_{\mathrm{SL}}(q)$, where $I_{\mathrm{colloid}}(q)$ is the scattered intensity from dilute, polydisperse, hard spheres and $I_{\mathrm{SL}}(q)$ is the scattered intensity from finite-sized *fcc* SLs. Although the NCs have surface ligands, the hard sphere approximation is a good one because the scattering from the Au NCs is much stronger than the scattering from the ligands, which are composed of lighter elements. In addition, any faceting of these NCs is not substantial enough to change the form of the measured SAXS patterns. Typically, $I_{\mathrm{colloid}}(q) = P(q)$, where $P(q)$ is the form factor for hard spheres of radius r and takes the form $P(q, r, \sigma, c_1) = c_1 \int_0^\infty |F(q, r)|^2 V(r)^2 P(r, \sigma) dr$, where $F(q, r) = 3\frac{\sin(qr) - qr\cos(qr)}{(qr)^3}$, $V(r)$ is the volume of the hard sphere, $P(r, \sigma)$ is a Gaussian probability distribution centered at r with standard deviation σ, and c_1 is a number directly proportional to the number of hard spheres. To compute $P(q)$ we use xrsdkit (https://github.com/scattering-central/xrsdkit). For the SL term, we use $I_{\mathrm{SL}}(q) = P(q, r, \sigma, c_2) S_{\mathrm{SL}}(q)$, where $S_{\mathrm{SL}}(q)$ is the structure factor for a finite-sized *fcc* SL. The structure factor describes the modulation in q of the scattered intensity due to the spatial arrangement of the NCs. We model $S_{\mathrm{SL}}(q)$ as the sum of a set of Lorentzian line shapes each centered on a respective Bragg peak of an *fcc* lattice (indexed by their Miller indices) and an additional q^{-4} term. The Lorentzian lineshape is defined as $S_{\mathrm{L}}(q, q_0, \Gamma) = \frac{1}{\pi}\frac{\frac{1}{2}\Gamma}{(q-q_0)^2 + (\frac{1}{2}\Gamma)^2}$, where q_0 is the center of the curve and Γ is the full width at half maximum (FWHM). There is an additional side peak that appears at slightly lower q than the *fcc* (111) peak that we also model with a Lorentzian lineshape and index with the letters lq. The final model for the SL structure factor is $S_{\mathrm{SL}}(q) = c_{\mathrm{lq}} S_{\mathrm{L}}(q, q_{\mathrm{lq}}, \Gamma_{\mathrm{lq}}) + c_{111} S_{\mathrm{L}}(q, q_{111}, \Gamma_{111}) + c_{200} S_{\mathrm{L}}(q, q_{200}, \Gamma_{200}) + c_{220} S_{\mathrm{L}}(q, q_{220}, \Gamma_{220}) + c_{311} S_{\mathrm{L}}(q, q_{311}, \Gamma_{311}) + c_{222} S_{\mathrm{L}}(q, q_{222}, \Gamma_{222}) + aq^{-4}$, where c_{lq}, c_{111}, c_{200}, c_{220}, c_{311}, and c_{222} are numbers that in principle depend on the Bragg peak multiplicity and Debye-Waller factors but are treated here as fit parameters as is a. We note that the crystal structure does not necessarily need to be known in advance in order to use this model. Lorentzian or other suitable line shapes can be used to fit the observable Bragg peaks wherever they occur in a SAXS pattern. If knowledge of the crystal structure is desirable (for example to determine lattice spacings), then it can usually be determined from the number and location of Bragg peaks in a SAXS pattern.[8] Our final fit function is $I(q, r, \sigma, c_1, c_2, c_{\mathrm{lq}}, q_{\mathrm{lq}}, \Gamma_{\mathrm{lq}}, c_{111}, q_{111}, \Gamma_{111}, c_{200}, q_{200}, \Gamma_{200}, c_{220}, q_{220}, \Gamma_{220}, c_{311}, q_{311}, \Gamma_{311}, c_{222}, q_{222}, \Gamma_{222}, a) = I_{\mathrm{colloid}}(q, r, \sigma, c_1) + I_{\mathrm{SL}}(q, r, \sigma, c_2, c_{\mathrm{lq}}, q_{\mathrm{lq}}, \Gamma_{\mathrm{lq}}, c_{111}, q_{111}, \Gamma_{111}, c_{200}, q_{200}, \Gamma_{200}, c_{220}, q_{220}, \Gamma_{220}, c_{311}, q_{311}, \Gamma_{311}, c_{222}, q_{222}, \Gamma_{222}, a)$. We explicitly fit for $r, \sigma, c_1, c_2, c_{\mathrm{lq}}, q_{\mathrm{lq}}, \Gamma_{\mathrm{lq}}, c_{111}, q_{111}, \Gamma_{111}, c_{200}, q_{200}, \Gamma_{200}, c_{220}, q_{220}, \Gamma_{220}, c_{311}, q_{311}, \Gamma_{311}, c_{222}, q_{222}, \Gamma_{222},$ and a. The fits are done according to a typical least-squares minimization. We seeded our fits by limiting the range of values for most parameters such as the locations of the Bragg peaks based on the locations expected for an *fcc* lattice, the NC size, and the NC size polydispersity to ensure robustness of the fitting procedure. We typically fit the logarithm of the intensities to assist the fitting procedure in capturing the details of smaller features at higher q values. For justification of this model to describe the scattering from finite-sized SLs, see **Figure S3** and associated text.

Power law fits to the SL (111) FWHM. In order to quantify the coarsening kinetics of the SL (111) FWHM in the experiments, we fit the late-time behavior to a power law, $FWHM \sim t^{-\alpha}$. We fit the data

in **Figure 2e** at late times to focus on the coarsening regime. Each of the power law exponents varies by 10% at most, depending on the time range over which the data is fit. This uncertainty is smaller than the relative differences between exponents obtained for the different experiments. To facilitate comparison of the different experiments, we chose to fit the data over similar time periods.

Simulations of self-assembly. Simulations of SL growth and annealing were performed with an underdamped Langevin dynamics in the NVT ensemble in a cubic periodic box using the LAMMPS software.[9] NCs were represented as 10976 spherical particles with pairwise volume exclusion interactions given by a Weeks-Chandler-Andersen (WCA) potential,[10]

$$u_{\mathrm{WCA}}(r) = \begin{cases} 4\epsilon_{\mathrm{LJ}}[(\frac{\sigma}{r})^{12} - (\frac{\sigma}{r})^{6}] + \epsilon_{\mathrm{LJ}}, & \text{if } r \leq 2^{\frac{1}{6}}\sigma \\ 0, & \text{otherwise} \end{cases}$$

where σ is the diameter of the NC, r is the center-to-center distance, and ϵ_{LJ} is the strength of the repulsion. Additionally, NCs interact also *via* a pairwise attractive short-range Morse potential, $u_{\mathrm{M}}(r) = u_0(e^{-2\alpha(r-r_0)} - 2e^{-\alpha(r-r_0)})$ where $r_0 = 1.23\sigma$ is the position of the minimum of the potential well, $\alpha = 10/\sigma$ is the inverse range, and u_0 denotes the well depth. The Morse potential has been truncated and shifted to zero at $r = 3\sigma$. At a given temperature T, the diffusive timescale for NCs is given by $\tau = \gamma\sigma^2/k_{\mathrm{B}}T$ where γ is the friction coefficient in Langevin dynamics, and k_{B} is Boltzmann's constant. We have chosen the density of NCs in the simulation box, mass of NCs, temperature and simulation timestep as $0.01/\sigma^3$, $\gamma^2\sigma^2/\epsilon_{\mathrm{LJ}}$, $\epsilon_{\mathrm{LJ}}/k_{\mathrm{B}}$ and $5 \times 10^{-5}\tau$, respectively. To compare to experimental timescales, we assume that the NCs follow Stokes' law of diffusion, with friction coefficient relating to the solvent viscosity η as $\gamma = 3\pi\eta\sigma$. We then use $\sigma = 4.5$ nm, $\eta = 0.876 \times 10^{-3}$ Pa-s, and $T = 300$ K to obtain $\tau = 0.18$ μs.

NCs were initially equilibrated in the gas phase at $u_0 = k_{\mathrm{B}}T$, before being adiabatically quenched to $u_0 = 2.5k_{\mathrm{B}}T$ and $u_0 = 2.8k_{\mathrm{B}}T$ for the shallow and deeper quench respectively. In the case of the shallow quench, nucleation at this well-depth is prohibitively slow. We thus used a spherical defect-free *fcc* crystal of size 200 NCs as a seed to start crystal growth and annealing. The seed was introduced by excavating a vacuum bubble of radius 1σ larger than that of the seed in the gas phase simulation, and randomly eliminating gas phase particles to conserve the total particle number. A seed of 100 NCs or smaller disperses into the gas phase. A seed of 200 NCs continues accruing particles till macroscopic size without nucleation of any second cluster in the box throughout the simulation. A seed of 500 NCs gives qualitatively similar results. In the case of the deeper quench, multiple metastable liquid droplets spontaneously nucleate and quickly freeze into crystals. The use of a seed was found irrelevant to the spontaneous nucleation of other clusters, so our reported results for the deeper quench simulation were not obtained with a seed.

Crystallinity calculation. We analyze simulated self-assembly trajectories by tracking crystalline order with Steinhardt-Nelson order parameters.[11] At each time frame, we define nearest neighbors to each NCs with a cutoff on the center-to-center distances, $|\mathbf{r}_i - \mathbf{r}_j| \leq 1.5\sigma$. We then compute the local bond-orientational order as

$$\psi_{6m}^{(i)} = \frac{1}{n_i} \sum_{j \in n_i} \left(\frac{\sum_{m=-6}^{6} q_{6m}^{(i)} q_{6m}^{(j)*}}{\sqrt{\sum_{m=-6}^{6} q_{6m}^{(i)} q_{6m}^{(i)*}} \sqrt{\sum_{m=-6}^{6} q_{6m}^{(j)} q_{6m}^{(j)*}}} \right)$$

where n_i represent neighbors of the i-th particle, and $q_{6m}^{(i)} = \frac{1}{n_i}\sum_{j \in n_i} Y_{6m}(\Phi_{ij}, \theta_{ij})$ with $Y_{lm}(\Phi_{ij}, \theta_{ij})$ as the lm- spherical Harmonic functions of the angular coordinates of the vector $\mathbf{r}_i - \mathbf{r}_j$. A particle was defined to be locally crystalline if either its $\psi_6^{(i)}$ parameter was above a cutoff of 0.7, or if its neighbor was locally crystalline. All locally crystalline particles are then classified into clusters of direct or indirect neighbors, and particles in clusters of size larger than a cutoff of 100 NCs are defined to be crystals. This cutoff serves to eliminate transient associations of gas-phase NCs that are still less than a critical nucleus size. All reported results about the crystallinity of NCs in the SL phase are computed only over NCs in these large dense locally ordered clusters.

Validity of model of SL structure factor. To test our model for the SL structure factor, we simulated a finite-sized *fcc* SL composed of 10976 NCs 4.5 nm in diameter and computed the structure factor, $S(q)$ (**Figure S3**). In an experimental scattering configuration, this is the structure factor that would be obtained from an isotropically oriented ensemble of such SLs. The structure factor has two main regimes in q: the

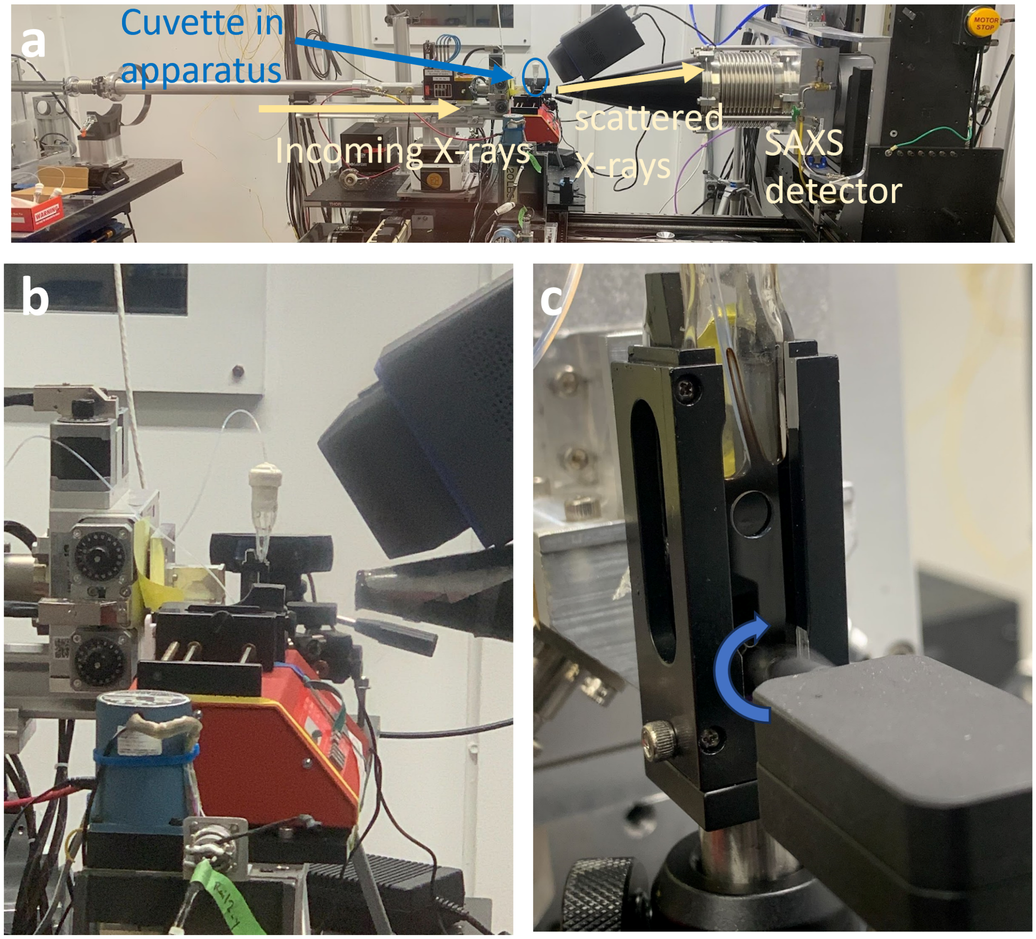

Figure S1: Experimental setup at SSRL beamline 1-5. (a) Zoomed out view of the apparatus at the beamline. X-rays are incident from the left. Scattered X-rays enter the SAXS flight tube and scatter onto the CCD detector on the right. (b) Close up picture of the apparatus at the beamline. (c) Face on view of the cuvette and stirrer. The circular recess in the cuvette indicates the location of the 200 μm window on the front face; a similar recess is found on the opposite side. The blue arrow indicates the direction of rotation of the magnetic stir bar in the cuvette.

Bragg scattering at high q and the scattering due to the finite size of the SL at low q. In principle, the scattering at low q from this single SL has oscillations with a $\sim q^{-4}$ envelope. These oscillations are difficult to see in **Figure S3** due to the limited q-resolution with which the structure factor was calculated in this region. These oscillations are not present in the experimental SAXS patterns despite them having greater q-resolution. The oscillations arise due to the finite size of the SLs, and their exact frequency and phase depend on the size of the SL. When there are many SLs of different sizes as there are in experiment, these oscillations wash out, leaving behind a smooth $\sim q^{-4}$ dependence, which is what is seen in the experimental SAXS patterns. A fit of our model to the structure factor of the simulated finite-sized *fcc* SL is shown in **Figure S3**. We note that we cut off the low-q term, aq^{-x}, at a q value before the appearance of the SL Bragg peaks. In the fitting shown here and in the main text, we chose $q = .1$ Å^{-1}, but the results of the fitting procedure are insensitive to the exact choice of this cutoff.

Volume fraction of the SL phase as a function of quench depth. To understand why the volume fraction of the SL phase increases as the quench depth increases, we first compute the nearest neighbor distance between NCs in the SL phase with the following procedure. From fitting the SAXS patterns of the colloidal NCs (**Figure 2a** main text), we extract a Gaussian NC size distribution of 4.5 ± 0.3 nm (**Figure**

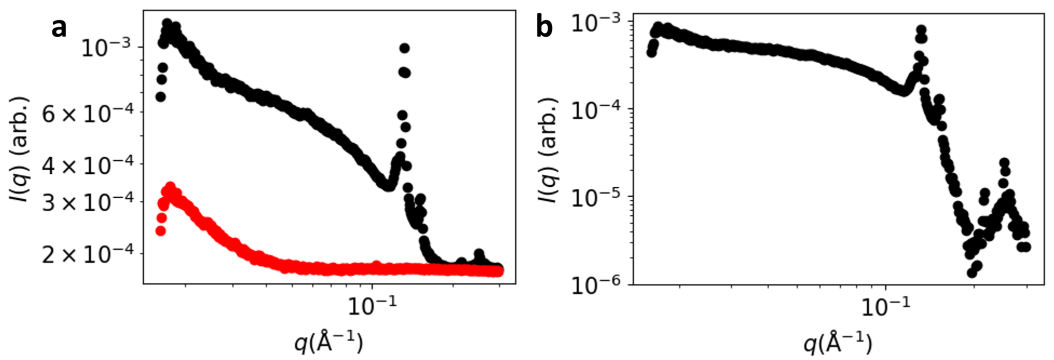

Figure S2: Background subtraction of SAXS patterns. (a) Single SAXS pattern taken from an *in situ* experiment (black dots) and from a background consisting of a cuvette filled with 1.1 M ionic strength salt in hydrazine (red). (b) SAXS pattern obtained after subtraction of the red curve from the black curve in (a). All SAXS patterns used in subsequent analysis were background subtracted in this way.

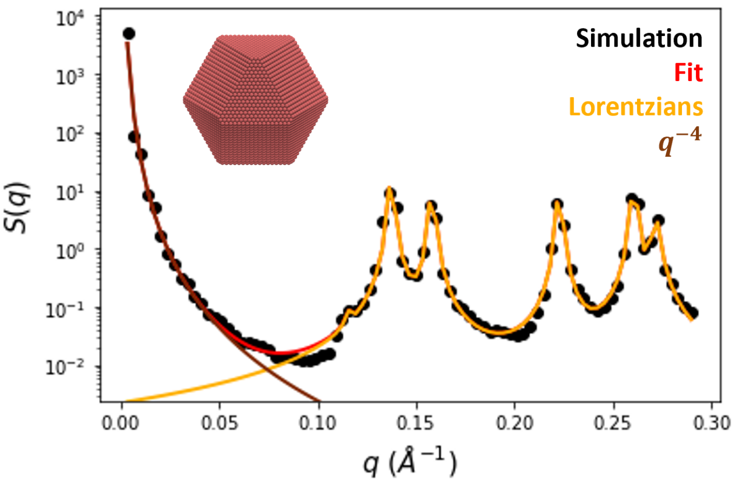

Figure S3: Structure factor and fit to structure factor of a simulated finite-sized *fcc* SL. Inset: real space structure of the *fcc* SL from which the structure factor was calculated.

S7a). From the fits to the SL *fcc* (111) Bragg peaks in the experimental and simulated SAXS patterns, we extract the SL lattice constants using $a = 2\pi\sqrt{3}/q_{111}$. We convert the lattice constants into center-to-center nearest neighbor distances by dividing a by 2 (**Figure S7b, S7c**). Using the extracted NC size distribution, we compute the surface-to-surface nearest neighbor distance as a function of time and quench depth (**Figure S7b, S7c**). We find that as a function of quench depth, the NCs in the deepest experimental quench are on average $\sim$2.8 Å closer together than the NCs in the shallowest experimental quench. One possible explanation for this trend is that at deeper quenches, the interactions are stronger and the particles are on average closer together. In the simulations, the NCs in the SLs in the deeper quench simulation are on average $\sim$ 0.8 Å closer together than the NCs in the SL in the shallow quench simulation.

Another possible explanation is that the NCs selectively quench depending on their size. In other words, the largest NCs, which experience the greatest van der Waals attraction, might incorporate into the SL phase before the smallest NCs do. To test this hypothesis, we performed a simple numerical test. First, we calculated the magnitude of the dispersion force between NCs as a function of their radii in **Figure S7d**. We find the dispersion force can vary by up to 1 $k_\mathrm{B}T$ at room temperature over the NC sizes present here. This variation can be substantial compared to the overall attractive force between NCs, which is $\sim$ several $k_\mathrm{B}T$.

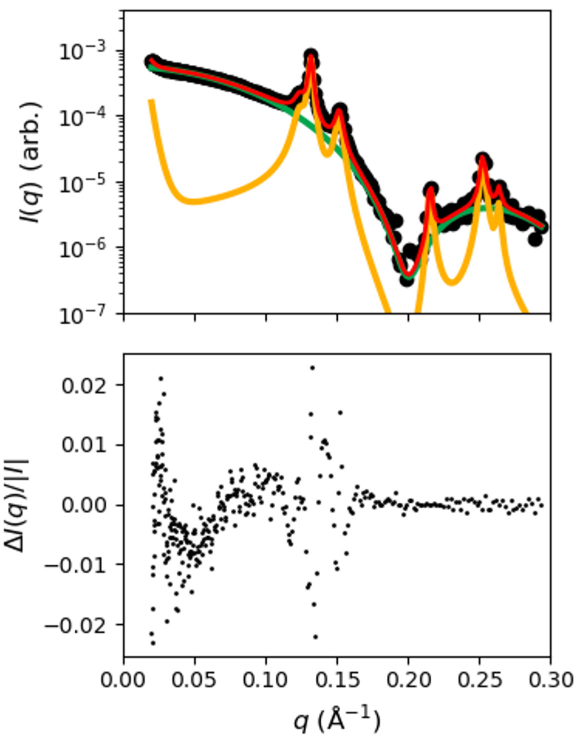

Figure S4: Residuals of a quantitative fit of model to a SAXS pattern. The residuals (data - fit) correspond to the fit to the SAXS pattern shown above. The residuals are normalized to the maximum intensity, $|I|$, of the SAXS pattern that was fit. The magnitude of the residuals is on the order of a few percent at worst and these deviations are largely centered around zero. The structure at low-q results from imperfect background subtraction (see form of background scattering in **Figure S2**). The discrepancy around the Bragg peak location occurs due to the peak not having a perfectly Lorentzian line shape. These slight discrepancies between the fitted model and the data do not limit the utility of the fit in quantifying the amount of NCs in the colloidal phase and the location and width of the (111) Bragg peak.

Second, to further test this hypothesis, we calculated the expected difference in nearest neighbor distances assuming that size selectivity occurs. At the shallowest quench depth, 60% of the original colloidal NCs end up in the SL phase at the end of our measurement, while at the deepest quench depth, 98% of the original colloidal NCs end up in the SL phase. If we assume the largest 60% and largest 98% of NCs selectively form SLs in each of those quenches, the average size of the 98% subpopulation is ~2.1 Å smaller than that of the 60% subpopulation. The magnitude and direction of this difference is consistent with the ~2.8 Å decrease in nearest neighbor distance found in **Figure S7b** when comparing the same respective quench depths. In conclusion, this polydispersity could account for a large portion of the difference in nearest neighbor distances between the shallowest and deepest quenches. We note that the SLs contract as a function of time across quench depths, decreasing by ~0.2 - 0.4 Å. These are very small changes that could be due to rearrangements of overlapping ligand shells on neighboring NCs.

Equilibrium NC fractions in colloidal and SL phases. We calculated the equilibrium volume fraction of the colloidal phase in **Figure 2c** in the main text based on the fraction of NCs in the colloidal phase ~1 hour post-quench. The colloidal NC fractions decrease by ~0.01-1% over a one hour time window starting after the initial quenches. Therefore, we estimate that the colloidal NC fractions we obtained ~1 hour post-quench should be within a few percent of the true equilibrium colloidal NC fractions. We calculated the volume fraction of the SL phase based on the q value on which the SL *fcc* (111) peak is centered for SLs ~1 hour post-quench. The SLs ~1 hour post-quench have not reached equilibrium. Deviations from equilibrium include the finite-size of the SLs and any strain/disorder in the SLs. These deviations from

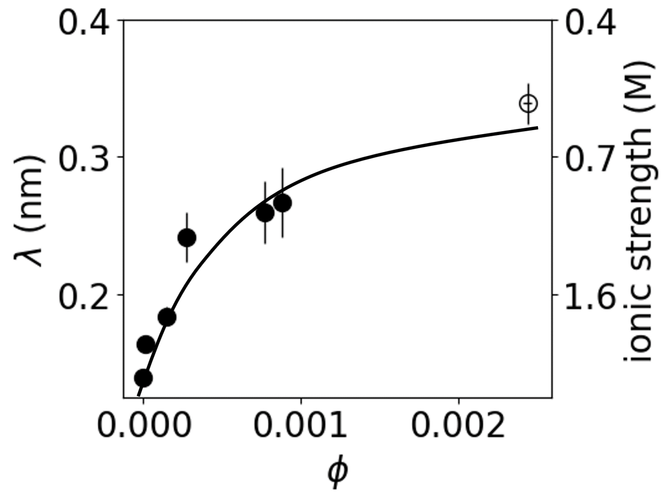

Figure S5: Close-up of low density binodal of the phase diagram extracted from experiments. Error bars are derived as in **Figure 2c** of the main text. The open circle indicates a (ϕ, λ) phase diagram location in which no SL formation was observed. The black phase boundary curve is drawn in as a visual guide. The open circle bounds the height of the phase boundary curve at $\phi \sim 0.0024$.

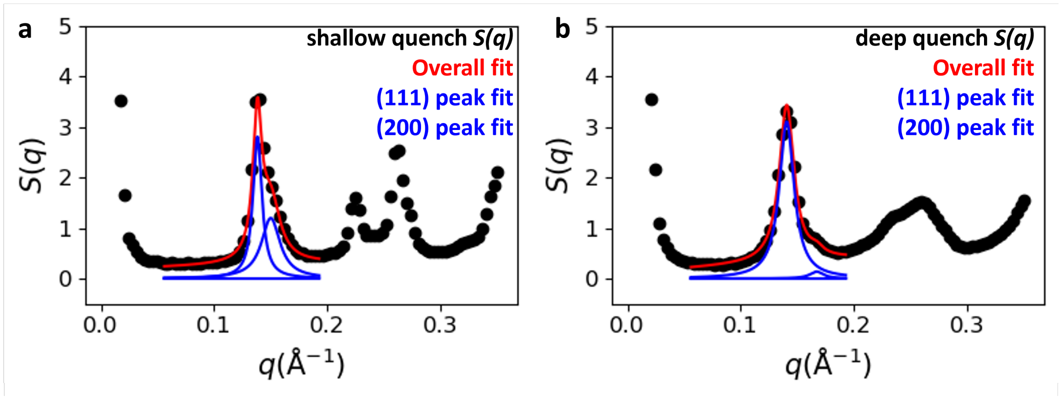

Figure S6: Structure factors and fits of the particles in the simulations. (a) Shallow quench structure factor and fit. (b) Deeper quench structure factor and fit.

equilibrium primarily contribute to the FWHM of the Bragg peaks and not the locations of the Bragg peaks. As a result, we expect that the volume fraction of the SL phase that we extracted to be a good estimate of the equilibrium SL volume fraction. One exception to this hypothesis is isotropic strain: SLs with isotropic strain have Bragg peaks shifted to lower q values than those of unstrained SLs and higher order Bragg peaks are more affected than lower order Bragg peaks.[12] The SLs formed in this work have Bragg peaks that are well indexed to those of an *fcc* crystal, indicating that any strain in the SLs is likely not isotropic in nature. Another possible exception is as follows: we might naively expect that for large amounts of disorder, the density of the condensed phase would decrease as close packing is no longer achieved, *i.e.*, a lower-density gel or aggregate forms instead of a SL. We are not in this regime for any of the experiments performed in this work. The more disordered SLs formed at deeper quenches, in fact, have *fcc* (111) peaks located at higher q values (more dense) than more ordered SLs formed at shallower quenches.

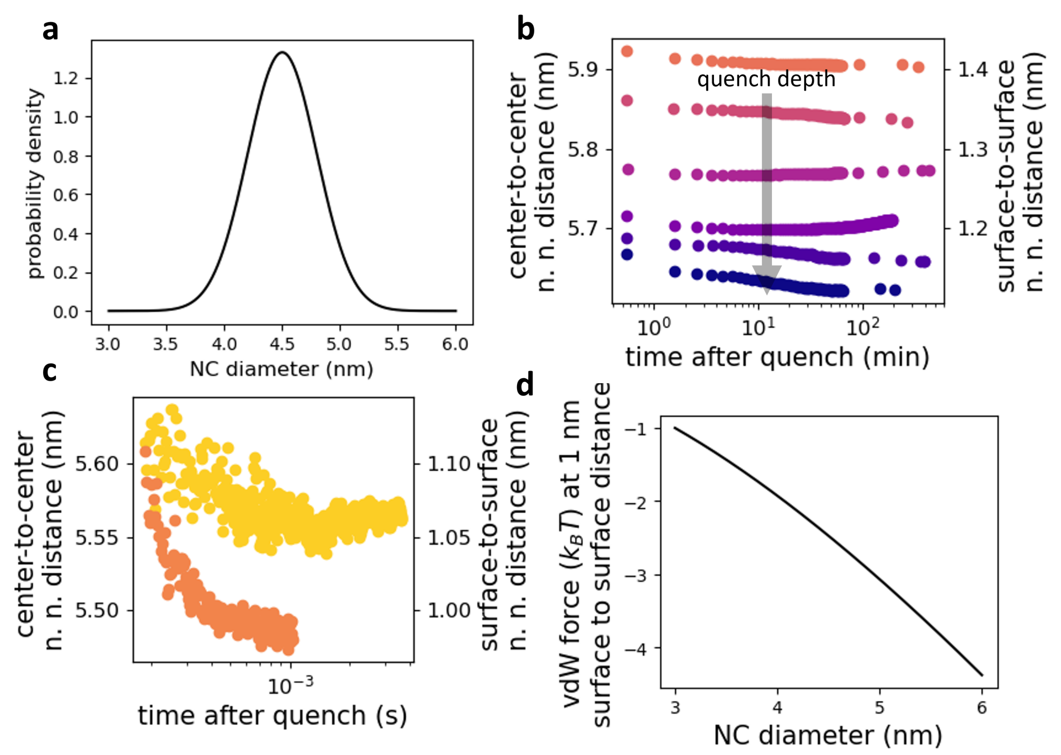

Figure S7: Size selectivity in NC SL self-assembly. (a) NC size probability distribution obtained from SAXS fitting. (b) center-to-center and surface-to-surface nearest neighbor distances between NCs in the SL phase as a function of time and quench depth in experiments. Color scheme is the same as **Figure 2** in the main text. (c) same as (b) except for NCs in the simulated SLs. (d) van der Waals force between NCs at a surface-to-surface separation of 1 nm as a function of NC diameter.

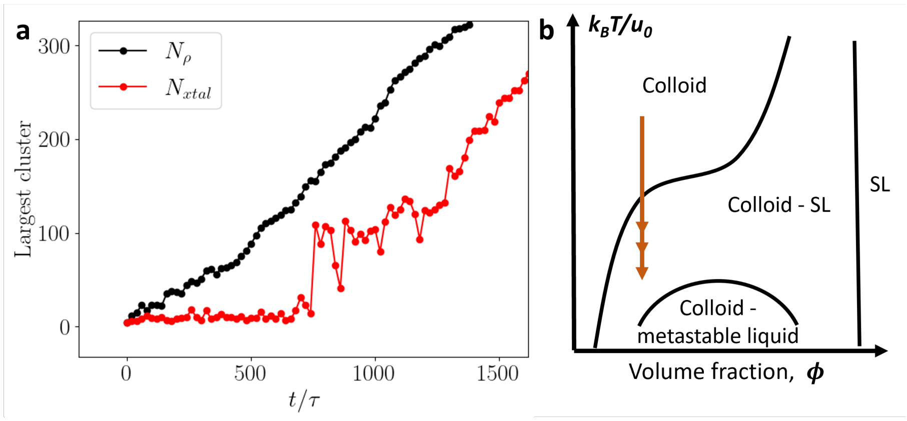

Figure S8: Short-lived or absent metastable liquid phase in *in situ* SAXS data. (a) Number of NCs in dense (black points) and crystalline (red points) phases for the largest cluster in the deeper quench simulation. See **Methods** section of the main text for definition of τ. SL nucleation occurs between 500-1000τ or 100-200 μs. (b) Schematic phase diagram of particles interacting with short-range interactions. The colloid-metastable liquid binodal drawn here peaks at lower k_BT/u_0 than the binodal in **Figure 2b**. The brown arrows indicate schematic quenches from the colloid to colloid-SL coexistence regions.

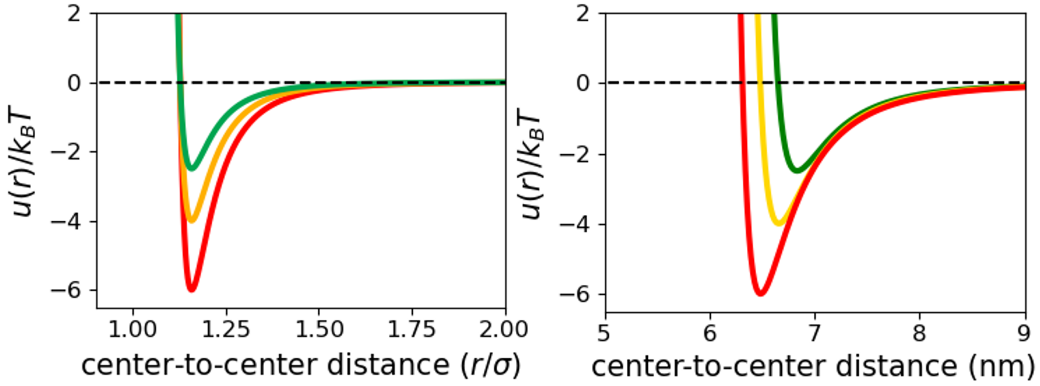

Figure S9: Interparticle potentials for shallow, intermediate, and deep quenches. Interparticle potential, $u(r)$, between two NCs as a function of their center-to-center distance parameterized relative to the effective size of the NCs (left) and in nm (right). Shallow quenches are shown in green, intermediate quenches are shown in gold, and deep quenches are shown in red. The depths of the potential were chosen to span the $\sim$2.5 - 6 $k_{\mathrm{B}}T$ range inferred in the main text. The range of the potentials was approximated to be $\sim$20% of the effective NC size for each quench depth. We then assume the effective size of the NCs corresponds to the center-to-center distance between NCs in the SL phase in **Figure S7b**. We multiply the center-to-center distance in r/σ by the effective size of the NCs to obtain the range in nm.

References

(1) Zhang, H.; Dasbiswas, K.; Ludwig, N. B.; Han, G.; Lee, B.; Vaikuntanathan, S.; Talapin, D. V. Stable Colloids in Molten Inorganic Salts. *Nature* **2017**, *542*, 328–331.

(2) Kamysbayev, V.; Srivastava, V.; Ludwig, N. B.; Borkiewicz, O. J.; Zhang, H.; Ilavsky, J.; Lee, B.; Chapman, K. W.; Vaikuntanathan, S.; Talapin, D. V. Nanocrystals in Molten Salts and Ionic Liquids: Experimental Observation of Ionic Correlations Extending beyond the Debye Length. *ACS Nano* **2019**, *13*, 5760–5770.

(3) Silvera Batista, C. A.; Larson, R. G.; Kotov, N. A. Nonadditivity of Nanoparticle Interactions. *Science* **2015**, *350*, 1242477.

(4) Kovalenko, M. V.; Scheele, M.; Talapin, D. V. Colloidal Nanocrystals with Molecular Metal Chalcogenide Surface Ligands. *Science* **2009**, *324*, 1417–1420.

(5) Guerrero García, G. I.; Olvera de la Cruz, M. Polarization Effects of Dielectric Nanoparticles in Aqueous Charge-Asymmetric Electrolytes. *The Journal of Physical Chemistry B* **2014**, *118*, 8854–8862.

(6) Coropceanu, I. et al. Self-assembly of Nanocrystals into Strongly Electronically Coupled All-Inorganic Supercrystals. *Science* **2022**, *375*, 1422–1426.

(7) Biggs, S.; Mulvaney, P. Measurement of the Forces between Gold Surfaces in Water by Atomic Force Microscopy. *The Journal of Chemical Physics* **1994**, *100*, 8501–8505.

(8) Warren, B. *X-ray Diffraction*, 2nd ed.; Dover Publications, Inc.: NY, 1990.

(9) Thompson, A. P.; Aktulga, H. M.; Berger, R.; Bolintineanu, D. S.; Brown, W. M.; Crozier, P. S.; in 't Veld, P. J.; Kohlmeyer, A.; Moore, S. G.; Nguyen, T. D.; Shan, R.; Stevens, M. J.; Tranchida, J.; Trott, C.; Plimpton, S. J. LAMMPS - a Flexible Simulation Tool for Particle-Based Materials Modeling at the Atomic, Meso, and Continuum Scales. *Computer Physics Communications* **2022**, *271*, 108171.

(10) Weeks, J. D.; Chandler, D.; Andersen, H. C. Role of Repulsive Forces in Determining the Equilibrium Structure of Simple Liquids. *The Journal of Chemical Physics* **1971**, *54*, 5237–5247.

(11) Steinhardt, P. J.; Nelson, D. R.; Ronchetti, M. Bond-Orientational Order in Liquids and Glasses. *Physical Review B* **1983**, *28*, 784–805.

(12) Khorsand Zak, A.; Abd. Majid, W. H.; Abrishami, M. E.; Yousefi, R. X-ray analysis of ZnO nanoparticles by Williamson–Hall and size–strain plot methods. *Solid State Sciences* **2011**, *13*, 251–256.
